# Short‐Form Video Media Use Is Associated With Greater Inattentive Symptoms in Thai School‐Age Children: Insights From a Cross‐Sectional Survey

**DOI:** 10.1002/brb3.70656

**Published:** 2025-07-07

**Authors:** Romteera Chiencharoenthanakij, Kachawan Yothamart, Naphat Chantathamma, Worachot Sukhumdecha, Saranyu Charoensri, Bhupa Thanyakulsajja, Krittisak Anuroj

**Affiliations:** ^1^ Department of Psychiatry, Faculty of Medicine Srinakharinwirot University Nakhon Nayok Thailand; ^2^ Faculty of Medicine Srinakharinwirot University Nakhon Nayok Thailand

**Keywords:** attention, digital media, media exposure, school‐age population, Thai

## Abstract

**Background:**

Short‐form videos, characterized by fast‐paced and high‐arousal content, may have unique effects on children's attention development distinct from other forms of media. However, their impacts remain underexplored, especially in school‐age children, a critical period for prefrontal circuitry's maturation.

**Objective:**

This study examined the association between short‐form video media use and inattentive behaviors among school‐age children, drawn from both clinical and nonclinical samples, while also exploring the associations with hyperactive–impulsive and oppositional‐defiant behaviors as secondary outcomes.

**Designs, Settings, and Participants:**

A cross‐sectional survey was conducted at a tertiary hospital in Thailand between November 2023 and March 2024. Participants included guardians of children aged 6–12 attending outpatient vaccinations and child psychiatric clinics. Inattentive behaviors and secondary outcomes were assessed using the parent‐rated Thai version of the SNAP‐IV short form. Generalized linear models examined their associations with short‐form video media use, adjusted for covariates such as total screen time, demographic data, parenting practices, and parental mental wellbeing.

**Results:**

The analysis included 528 participants, with 11.6% previously diagnosed with ADHD. Short‐form video media use was significantly associated with higher inattentive behaviors. The association was stronger among younger participants. No significant associations were found with hyperactive–impulsive or oppositional‐defiant behaviors, although total screen time remained significantly associated with these outcomes.

**Conclusion:**

The observed association between short‐form video media use and inattention, together with the significant negative interaction with age in this school‐age sample, adds to the growing literature on the neuropsychiatric impacts of the media and underscores the needs for studies on mechanisms and long‐term impacts.

## Graphical abstract

This survey examined the relationship between short‐form video use and attention in school‐age children. Analyzing responses from 528 participants, short‐form video use was linked to higher inattentive symptoms, with a stronger association in younger children. Further studies on the long‐term effects and potential bidirectional mechanisms are needed.

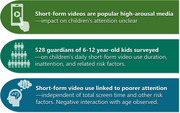

**Abbreviations**
ADHD: attention‐deficit/hyperactivity disorder
PERMA: positive emotion, engagement, relationships, meaning, and accomplishment
SNAP‐IV: Thai version of Swanson, Nolan, and Pelham IV scale

## 1 Background

Attention is a fundamental neurocognitive function that governs the selective processing of sensory inputs, thoughts, and tasks. It plays a crucial role in children's development, particularly in the acquisition of knowledge, skills, and problem‐solving abilities (Berk 2018). Genetic predispositions, prenatal neurodevelopmental influences, and environmental factors such as socioeconomic status, stimulation, and adverse life experiences are among the important factors affecting the development of attentional capacity (Balázs and Keresztény 2014; Vogel et al. 2018). Although attentional capacity increases with age, in a given age group, the capacity is distributed continuously from low to high across the population (Sonuga‐Barke et al. 2015). Clinically significant attention disturbances—often accompanied by hyperactivity and impulsivity—are not considered as a discrete entity but rather a quantitative trait that falls at the extreme end of this distribution, classified as attention‐deficit/hyperactivity disorder (ADHD) (Sonuga‐Barke et al. 2015; Greven et al. 2016). Yet, the continuous distribution means that subthreshold inattentive symptoms are also widespread in the general population (Vogel et al. 2018). Interestingly, falling below the diagnostic threshold does not preclude negative outcomes. Research has shown that children with subclinical inattentive symptoms are at increased risk for future academic and occupational difficulties, addiction behaviors, mental health problems, and cognitive impairments, albeit to a lesser extent than those with a clinical diagnosis (Vogel et al. 2018; Schiavone et al. 2024; Vörös and Lukovszki 2021; Lundervold et al. 2017; Gostoli et al. 2024; Tengsujaritkul et al. 2020; Jaisoorya et al. 2019). Coinciding with these observations, the *Diagnostic and Statistical Manual of Mental Disorders*, Fifth Edition (DSM‐5) has begun to acknowledge dimensionality to some extent, and alternative frameworks adopting a dimension‐oriented approach have emerged in recent years—many of which place greater emphasis on subthreshold symptoms (American Psychiatric Association 2022; Kotov et al. 2017; Musser and Raiker 2019). In light of these findings and developments, inattentive problems—manifesting across a spectrum of behaviors, symptoms, and functional disturbances—are viewed within this study's framework as part of a continuum, rather than solely as part of a specific disorder.

Attention is a coordinated function of multiple brain regions that undergo continuous development from the fetal period through birth and into adolescence. Children's attentional capacity expands considerably as they transition from kindergarten to the more demanding environment of primary school around the ages of 6–7 (Berk 2018; Williams et al. 2019; Betts et al. 2006). The transition also marks the period where attention‐related difficulties related to neurodevelopmental issues become more apparent, as differences from peers start to widen (Sonuga‐Barke et al. 2015). Fortunately, the prefrontal cortex, a key region involved in attention, continues to mature throughout childhood into adolescence through processes such as ongoing myelination and synaptic pruning (Tooley et al. 2022). With this developmental window, studying modifiable risk factors associated with attentional difficulties could offer opportunities for interventions to mitigate the divergence in brain development.

Screen media use is a potentially modifiable risk factor for attention problems, having become increasingly pervasive in daily life and the focus of active research. A bidirectional relationship has been suggested: screen media use may contribute to attention disturbances and alterations in related brain regions, while core features of ADHD—including the disturbances in attention—may increase susceptibility to excessive or problematic media use (Beyens et al. 2018; Morita et al. 2022). However, as media continuously evolve alongside technological advancements, the definition of “screen media use” has never remained static. Short‐form videos represent a relatively recent form of media that has gained significant popularity over the past decade. Initially popularized by the now‐defunct platform Vine, short‐form videos have since become ubiquitous across social media platforms, appearing under various names such as Shorts (on YouTube), Reels (on Facebook and Instagram), or simply as videos (on TikTok). These videos are typically only seconds long—usually no longer than a minute—and offer a wide variety of content. Short‐form videos are inherently high‐arousal as their core content is condensed and delivered within a brief timeframe. The continuous, scroll‐based navigation—made possible by modern internet infrastructure—differs from traditional browsing or searching methods and may reinforce a preference for immediate gratification, particularly appealing to children's limited attention spans (Qin et al. 2022; Yan et al. 2023; Jiang and Yoo 2024). In addition, video site's algorithms quickly adapt to children's preferences for more high‐arousal content, curating a narrowed selection of videos for them to engage with (Qin et al. 2022; Zhao 2021). Understanding the relationship between this specific form of media and inattentive symptoms may offer updated insights to guide future research and inform clinical practices as well as public health recommendations.

### 1.1 Objective

The primary objective of this study is to examine the relationship between daily duration of short‐form video media use and parent‐rated inattentive behaviors in school‐age children. The secondary objectives are to explore the relationship between short‐form video media use and hyperactive–impulsive and oppositional‐defiant behaviors.

## 2 Material and Methods

### 2.1 Design and Settings

This is a cross‐sectional study conducted in a suburban tertiary hospital between November 2023 and March 2024. Participants included literate guardians, aged 19–60, of children aged 6–12, attending outpatient vaccinations or child psychiatric clinic visits. Given the limited accessibility of mental health services in Thailand, some children in the general population sample may have undiagnosed attention difficulties, while others could have milder or minimal symptoms. The two designated settings aimed to capture a diverse sample from both clinical and general population settings, reflecting the full spectrum of inattentive behaviors—from minimal and subthreshold symptoms to clinically significant ones. All eligible guardians of children visiting within the study period were approached for participation. Data were collected once using guardian‐rated questionnaires.

Children with guardian‐reported diagnoses of global developmental delay, intellectual disability, or autism spectrum disorder were excluded, as their conditions were expected to distort ratings of inattentive behaviors.

Given the prevalence of skip‐generation parenting in the context of the studied population, ratings from extended family members acting as de facto guardians—who were familiar with the child's behaviors through active parental care—were also permitted.

### 2.2 Participants

A total of 537 guardians participated in the study; however, nine participants from vaccination clinics were excluded due to high outlier data in the screen time data, resulting in a final sample of 528 participants for the analysis. Of these, 73 (13.8%) were from the psychiatric clinic, and 455 (86.2%) were from routine pediatric vaccination visits. Children with psychiatrist‐diagnosed ADHD under the DSM‐5 system, who represented the extreme end of the attention continuum (Sonuga‐Barke et al. 2015; Greven et al. 2016), comprised approximately 12% of the sample. Characteristics of the participants and children are presented in Table 1.

**TABLE 1:** Demographic characteristics and SNAP‐IV ratings of the sample.

	*N* (valid %) or Mean (SD)
Source of participants Psychiatric clinic Vaccination clinic	73 (13.8%) 455 (86.2%)
Guardian's relationship with children Parents Extended family	410 (77.7%) 118 (22.3%)
Guardian's gender Male Female	133 (25.2%) 395 (74.8%)
Religion Buddhist Other	462 (87.5%) 66 (12.5%)
Guardian's age (year)	38.9 (8.4)
Guardian's employment status Governmental sector Private sector Unemployed/full‐time parents	190 (36.0%) 274 (51.9%) 64 (12.1%)
Guardian's educational level Bachelor's degrees and above High school Below high school	203 (38.4%) 238 (45.1%) 87 (16.5%)
Familial financial status Secure with savings Borderline Indebted	149 (28.2%) 236 (44.7%) 143 (27.1%)
Perinatal/prenatal complications Gestational diabetes Preterm birth Preeclampsia Others	19 (3.6%) 10 (1.9%) 4 (0.8%) 19 (3.6%)
Child's gender Male Female	303 (57.4%) 225 (42.6%)
Child's age (year)	9.2 (2.1)
Psychiatric comorbidities ADHD Other psychiatric diagnoses^a^	61 (11.6%) 24 (4.5%)
Medical conditions Allergies Asthma Others	26 (4.9%) 4 (0.8%) 21 (4.0%)
Daily screen time (h) Total Viewing short‐form videos	3.6 (2.2) 1.9 (1.4)
SNAP‐IV ratings Inattentive behaviors Hyperactive–impulsive behaviors Oppositional defiant symptoms behaviors	9.0 (5.7) 6.6 (5.4) 6.2 (5.1)

Abbreviation: SNAP‐IV: Swanson, Nolan, and Pelham IV scale—short form, Thai version.

^a^ Included 16 cases with specific learning disorder (10 comorbid with ADHD), 5 with anxiety (1 comorbid with ADHD), 2 with depression (1 comorbid with ADHD), and 1 with tics.

### 2.3 Sample Size

The sample size was determined prior to the survey using G*Power version 3.1.9.7 (Faul et al. 2007; Faul et al. 2009). To achieve a power of 0.9 and an alpha level of 0.05 in detecting a small effect size (*f* ^2^ = 0.04, as converted from the observed effect size of overall screen media use) of short‐form video media use in predicting ADHD symptoms in linear regression, a total of 485 participants was required (Beyens et al. 2018; Boer et al. 2020).

### 2.4 Measurements and Variables

A parent‐rated scale was chosen to assess inattentive behaviors, as children at this age have limited introspective capacity. In Thailand, school‐age children are typically under close supervision outside of school hours by their parents or relatives designated as guardians, making them knowledgeable informants for children's inattentive behaviors in real‐world settings. Inattentive behaviors were measured using the parent‐rated Thai version of Swanson, Nolan, and Pelham IV scale—short form (SNAP‐IV). The scale measures manifest behaviors in the inattentive, hyperactive–impulsive, and oppositional defiant domains on a Likert scale of intensity. The scale exhibited validity and reliability in both general and clinical populations, has been widely adopted in Thai clinical and research practices, and was deemed suitable for assessing inattentive behaviors in the specified population (Pityaratstian et al. 2014; Choopun and Boonlue 2022). For children receiving treatment for ADHD, typically with immediate‐release methylphenidate due to its cost and availability in Thailand, guardians were asked to rate symptoms during the medication's “off” periods or drug holidays so that the ratings reflect their baseline behaviors. The summed score of each domain was analyzed as a continuous variable. The subscales exhibited good internal consistency, with Cronbach's *α* values of 0.92 for the inattentive subscale, 0.91 for the hyperactive–impulsive subscale, and 0.92 for the oppositional defiant subscale.

Guardians were then asked to estimate their children's total daily screen media use (i.e., time spent on activities such as watching videos, playing games, and using social media) and short‐form video use separately, reported in hours. Most responses were in whole hours, with some including half‐hour increments. Estimates were provided for both average weekday and weekend uses to account for variation during the school semester. Daily averages for total screen time and short‐form video use were then calculated for subsequent analyses. As noted in Table 1, children spent an average of 3.6 h per day on screen use, with short‐form video viewing comprising approximately half (56.8 ± 30.6%) of total screen time. Demographic data, child's medical history (prenatal/perinatal complications, sleep disturbances, and psychiatric and medical comorbidities), parenting practices, and guardian's mental wellbeing were collected as potential covariates (Sonuga‐Barke et al. 2015; Freitag et al. 2012; Sagiv et al. 2013).

Parenting practices were measured using the Thai version of the Alabama Parenting Questionnaire, which assesses domains such as inconsistent parenting and poor monitoring, rated on a Likert scale of frequency. The Thai version had established convergence with ADHD symptoms (Khamon et al. 2019). Summed scores from each subdomain—inversed as appropriate so that higher scores reflected more optimal practices—were analyzed as continuous variables. The scale demonstrated high internal consistency (*α* = 0.91).

Guardian's mental wellbeing was measured using the PERMA profiler, which evaluates the guardian's life situation across the domains of positive emotion, engagement, relationships, meaning, and achievement, using bipolar anchored scales (Seligman 2011; Schrank et al. 2014; Butler and Kern 2016). The total score was analyzed as a continuous variable. The scale demonstrated high internal consistency (*α* = 0.90).

### 2.5 Statistical Analysis

Statistical analysis was conducted using SPSS version 27. The associations between daily average hours of short‐form video media use and inattentive behaviors, hyperactive–impulsive behaviors, and oppositional‐defiant behaviors were analyzed using generalized linear models with a robust estimator to provide more reliable standard error estimates in cases of minor model assumption violations. Variables were entered into the model and subjected to stepwise removal. Models were constructed separately for each dependent variable. Visual inspection of histograms showed that SNAP‐IV subscales followed continuous, right‐skewed distributions resembling gamma curves, without extreme outliers—patterns that supported the dimensional rather than dichotomous view of the symptoms. Analysis of Q–Q plots confirmed that the distribution of SNAP‐IV subscales approximated the gamma distributions, which was accordingly specified in the generalized linear models along with the canonical log link function. In addition to main effect analyses, interaction terms between short‐form video media use and covariates were added to the models in an exploratory manner.

Missing data were handled using multiple imputations. Most cases with missing data involved a single missing variable. The rate of missing data across variables ranged from 0% to 2.1%, all due to guardian's non‐response. The highest missingness occurred in one of the hyperactivity items on the SNAP‐IV questionnaire, with no discernible pattern or apparent systematic cause. Given the low overall rate, multiple imputation was used to preserve sample size and minimize potential bias.

## 3 Results

The generalized linear model revealed a significant positive association between short‐form video media use and inattentive behaviors, independent of total screen time and demographic and familial covariates (see Table 2’s footnote), with a McFadden's pseudo *R* ^2^ of 0.08. Each one hour increase in short‐form video viewing increased inattentive behaviors by a factor of 1.04. Total screen time possessed a positive relationship with inattentive behaviors in the model as well. Further exploration of interactions revealed a significant negative interaction between short‐form video media use and child's age. Parameter estimates are shown in Table 2

**TABLE 2:** Regression coefficients and their exponentiated values of short‐form video media use (in hour) from generalized linear models predicting inattentive, hyperactive–impulsive, and oppositional‐defiant behaviors.

	Simple regression			Covariate‐adjusted^a^		
	*B* coefficient	Exp (*B*)	*p* value	Adjusted coefficient^a^	Exp (B)	*p* value
**Inattentive behavior: main effect**						
Short‐form video media use	0.08	1.08	< 0.01	0.04	1.04	< 0.01
**Inattentive behavior: interaction**						
Short‐form video media use * child's age				−0.01	0.99	0.02
**Hyperactive–impulsive behaviors: main effect**						
Short‐form video media use	0.06	1.05	< 0.01	0.00	1.00	0.82
**Oppositional‐defiant behaviors: main effect**						
Short‐form video media use	0.08	1.08	< 0.01	0.02	1.02	0.16

^a^ Controlled for covariates, including guardian's education level, financial status, mental well‐being, and parenting practices, as well as children's age, gender, physical and psychiatric comorbidities, and total screen time, with backward eliminations.

Gamma distribution with a canonical log link function was specified for all models. Exp (*B*) converts the coefficients into interpretable effect sizes, representing the multiplicative changes in the outcome for each unit increase in the predictor.

The dependent inattentive, hyperactive–impulsive, and oppositional defiant behaviors represent summed ratings from the Thai version of the SNAP‐IV short form.

Short‐form video media use did not show statistically significant associations with hyperactive–impulsive or oppositional‐defiant behaviors in their respective models (McFadden's pseudo *R* ^2^‐square of 0.09 and 0.12), although total screen time remained significantly positively associated with these behaviors.

## 4 Discussion

This study analyzed a sufficiently large, mixed clinical and nonclinical sample of Thai school‐age children and observed the expected continuous distribution of inattentive behaviors (Sonuga‐Barke et al. 2015). The analyses accounted for total screen time and other known proximal risk factors for inattentive and hyperactive–impulsive symptoms, such as parenting practices and guardian's mental health (Sonuga‐Barke et al. 2015; Freitag et al. 2012; Sagiv et al. 2013). The results provide preliminary evidence for an association between short‐form video media use and attention problems, aligning with recent findings—such as a study linking short‐form video addiction tendencies with reduced prefrontal theta power, and another study in adults identifying mediating relationships between ADHD symptoms and problematic short‐form video use (Yan et al. 2024; Xu et al. 2025). The pseudo *R* ^2^ values in our study reflected a relatively low improvement in model fit compared to the null model, which may be attributed to both the multifactorial nature of attention development and the relatively small effect size of short‐form video media use. However, the statistics should not be interpreted as a direct reflection of variance explained, as with ordinary least squares *R* ^2^. The limited model fit, however, underscores the need for future models investigating ADHD symptoms to carefully incorporate variables and interactions to effectively capture their variances.

As observed in individuals with ADHD and their subclinical counterparts, attention difficulties may predispose individuals to addictive behaviors (Gostoli et al. 2024; Karaca et al. 2017; Md Yusop et al. 2024; Leung and Chan 2017). This vulnerability can be attributed to their heightened reliance on external stimuli to maintain arousal, boredom proneness, and dysfunctions in self‐monitoring, self‐regulation, decision making, motivation systems, and sensory processing (Xu et al. 2025; Shiels and Hawk 2010; Kleberg et al. 2023; Korman et al. 2019; Halbe et al. 2025; Bhaijiwala et al. 2014). By default, children may also revert to addictive behaviors after disengaging from other attention‐consuming tasks—a phenomenon explained by the exploration–exploitation model (Baumgartner et al. 2024). Coupled with their limited attentional capacity, these predispositions may drive children toward the high‐arousal, addictive qualities of screen media (Fikkers and Piotrowski 2020; Geissler et al. 2014). Short‐form videos, being both socially pervasive and inherently stimulating, are therefore prone to be overused by children with attention difficulties through self‐reinforcing cycles (Zhao 2021). The genre of consumed media may also play a role in interacting with personal characteristics to produce overuse (Kim et al. 2022; Flayelle et al. 2019), although this was not assessed in the current parent‐rated questionnaire due to challenges in obtaining reliable data. The difficulties partly arose from the brief and fragmented nature of video content, the prevalent use of small mobile devices for viewing, and generational gaps in recognizing or categorizing media genres.

Certain characteristics of short‐form video consumption—such as rapid pacing, gesture‐based continuous browsing, repetitive exposure to algorithm‐fed, brief, high‐arousal content, and the ability to multitask across platforms—resemble features of media previously linked to the development or exacerbation of attentional impairments (Zhao 2021; Christakis et al. 2018; Rioja et al. 2023). Experimental studies have shown that such media, especially those with fantasy elements, can immediately disrupt children's attentional performance, with cumulative effects also possible (Lillard and Peterson 2011; Lillard et al. 2015; Namazi and Sadeghi 2024). Unsurprisingly, long‐term effects on the development of attention and related cognitive functions have also been attributed to media use (Baumgartner et al. 2024; Christakis et al. 2018). This is likely driven by multiple interacting mechanisms, including cumulative cognitive overload, depletion of executive function capacity, dysregulation of arousal and reward systems, conditioning to quick, high‐arousal rewards, and media‐related sleep disturbances; together, these factors could strengthen the brain's bottom–up, stimulus‐driven circuitry while weakening prefrontal networks essential for executive function (Baumgartner et al. 2024; Marino et al. 2019; Ra et al. 2018; Thorell et al. 2022; Zhao et al. 2022; Zhao et al. 2023; He et al. 2023; Supanitayanon et al. 2020). Emotion dysregulation, closely linked to attention difficulties, may also interact with the Thai cultural preference for emotion suppression (Chiencharoenthanakij et al. 2024; Wungvivatchareon and Anuroj 2025) and further disrupts the prefrontal circuitry (Beheshti et al. 2020; Jacob et al. 2016). Additionally, opportunity cost of excess media use means that children spend less time on developmentally beneficial activities such as outdoor plays and face‐to‐face social interactions (Best 2010; Song et al. 2023; Chen 2024; Tenenbaum et al. 2020). These potential bidirectional mechanisms related to specific characteristics of short‐form videos could account for the media's statistical effect independent of total screen time. Further research is warranted to explore these bidirectional pathways and long‐term consequences, thereby informing evidence‐based clinical practice and public policy development.

Age appears to negatively moderate the association between short‐form video media use and inattentive behaviors, meaning that the association is stronger in younger children. This suggests that younger children may be more vulnerable to the effects of short‐form videos, potentially due to their developing brains being more susceptible to environmental stimuli (Whittle et al. 2025; Thompson and Steinbeis 2020). This aligns with current recommendations for limiting media exposure in younger children (Council on Communication and Media 2016). Should future studies confirm independent associations between short‐form video use and inattention, and if short‐form videos remain a significant part of children's screen time, it may be necessary to refine guidelines to include specific recommendations for this type of media. Beyond public health recommendations, familial behaviors—ranging from strict adherence to these guidelines to modeling media overuse due to shared traits of inattention—can either mitigate or exacerbate children's media overuse and attention issues.

Unlike the association with inattentive behaviors, short‐form video media use was not significantly linked to hyperactive–impulsive or oppositional defiant behaviors in covariate‐adjusted models. However, total screen timeremained a significant predictor of both domains. It is possible that the unique features of short‐form videos do not directly contribute to the development of these behaviors, nor do they strongly appeal to traits associated with hyperactivity or oppositionality in a way that would drive increased engagement or overuse. Instead, aligning with findings in ADHD, overall media exposure—be it short‐form videos or other screen‐based activities—potentially played a more prominent role and acted as a suppressor in the models by accounting for the variance shared with short‐form video use (Thorell et al. 2022). Nevertheless, future research should routinely assess domain‐specific effects of novel forms of media to determine their influences on these correlated symptom clusters.

## 5 Limitations

This study has several limitations. First, inattentive behaviors were assessed using the parent‐rated Thai version of the SNAP‐IV short form. Although the expected continuous distribution was observed, the variance in inattentive behaviors, particularly among children with milder symptoms, may not have been fully captured by the scale. Recall bias could be more prevalent among parents of symptomatic children, while more subtle inattentive behaviors, which could be more apparent in school settings, might be missed if teachers’ inputs to guardians were limited. Further studies focusing on subthreshold symptoms may either opt for more detailed scales, such as those examining distinct domains of attention, or use direct attention measurement at the expense of resources. Second, while including both clinical and subclinical populations aligned with the dimensional framework of inattentive problems and produced the expected distribution, it also introduced heterogeneity into the sample. The medicated children, although having their baseline (“off” period) symptoms rated, may still have exhibited variances attributable to residual therapeutic effects and other interventions (Klein et al. 2024). Third, the limited availability of mental health services in Thailand could have resulted in the presence of undiagnosed neurodevelopmental disorders within the community. While efforts were made to exclude diagnosed patients, full screening was not methodologically feasible, and inadvertent inclusions of milder, undiagnosed cases could have influenced the observed variance. Forth, unmeasured covariates, such as genre of media consumed, household media use rules, sleep quality, and undiagnosed mental health conditions, could have accounted for the unexplained variance. Finally, the cross‐sectional, questionnaire‐based design limited the ability to infer causal relationships or explore underlying mechanisms.

## 6 Conclusion

This study observed a relationship between short‐form video media use and increased inattentive behaviors in Thai school‐age children, contributing to the growing body of literature on the media's neuropsychiatric impacts. While it could be inferred that specific characteristics of short‐form video media accounted for the associations in a bidirectional manner, further research is needed to confirm these findings and explore the underlying mechanisms and long‐term consequences. The results also indicated a stronger association in younger children, aligning with current recommendations for limited screen time in this age group. Should future studies confirm a robust relationship between this form of media and attention problems, more specific guidelines may be warranted. No significant relationship was observed between short‐form video media use and hyperactive–impulsive or oppositional‐defiant behaviors, although total screen time remained significantly associated with these behaviors in their respective models.

## Author Contributions

**Romteera Chiencharoenthanakij**: conceptualization, methodology, investigation, funding acquisition, writing–original draft, writing–review and editing. **Kachawan Yothamart**: conceptualization, methodology, investigation, writing–original draft. **Naphat Chantathamma**: conceptualization, methodology, investigation, writing–original draft. **Worachot Sukhumdecha**: conceptualization, methodology, investigation, writing–original draft. **Saranyu Charoensri**: conceptualization, methodology, investigation, writing–original draft. **Bhupa Thanyakulsajja**: conceptualization, methodology, investigation, writing–original draft. **Krittisak Anuroj**: conceptualization, methodology, investigation, funding acquisition, validation, formal analysis, writing–original draft, writing–review and editing.

## Conflicts of Interest

The authors declare no conflicts of interest.

## Ethics Statement

The Human Research Ethics Committee of Srinakharinwirot University approved the study protocol (SWUEC‐662071).

## Consent

All participants provided written informed consent.

## Peer Review

The peer review history for this article is available at https://publons.com/publon/10.1002/brb3.70656

## Data Availability

The data analyzed during the current study are available from the corresponding author upon reasonable request.
